# Children’s Environmental Health in the Digital Era: Understanding Early Screen Exposure as a Preventable Risk Factor for Obesity and Sleep Disorders

**DOI:** 10.3390/children5020031

**Published:** 2018-02-23

**Authors:** Candice Wolf, Seth Wolf, Miriam Weiss, Gustavo Nino

**Affiliations:** 1 Larner College of Medicine, University of Vermont, Burlington, VT 05405-0068, USA; candice.wolf@med.uvm.edu (C.W.); seth.wolf@med.uvm.edu (S.W.); 2 Sleep Medicine Department, Children’s National Medical Center, Washington, DC 20010, USA; miweiss@childrensnational.org

**Keywords:** sleep, obesity, screen exposure, technology, pediatric, addiction, cognitive deficit, BMI, development

## Abstract

The quantity, accessibility and focus on child-targeted programming has exponentially increased since it entered American households in the early 1900s. It may have started with the television (TV), but technology has evolved and now fits in our pockets; as of 2017, 95% of American families own a smartphone. Availability and child-tailored content has subsequently led to a decrease in the age at initial screen exposure. The negative effects that accompany the current culture of early screen exposure are extensive and need to be considered as technology continues to enter the home and inundate social interactions. Increased levels of early screen exposure have been associated with decreased cognitive abilities, decreased growth, addictive behavior, poor school performance, poor sleep patterns, and increased levels of obesity. Research on the adverse effects of early screen exposure is mounting, but further epidemiological studies are still needed to inform prevention and regulation policies.

## 1. Introduction

Since television (TV) entered American homes in the late 1930s, parents have been concerned about the effect it could have on their children. The number of children-tailored programs has steadily increased; in 1951, there were 27 h per week directed towards children. By the 1980s, the cable industry made it possible for entire channels to be devoted to entertaining children 24/7 [1]. In the late 1990s, the first infant-directed TV programming was developed which has led to infants and toddlers consuming more TV than ever before [2,3]. As of 2007, most infants and toddlers actively watch 1–2 h of TV daily and are exposed to over three hours of additional background television [4,5]. In the 1970s, the average child began regularly watching TV around 4 years old, but in today’s media-rich climate, infants are exposed to digital media as early as 4 months of age [6]. The American Academy of Pediatrics recommends avoiding the use of screen media, other than for video chatting, for children 18 months and younger and limiting the amount for older children to less than one hour of daily quality programming [7].

As of 2011, children of all ages have slightly decreased their incidence of TV viewing, because mobile devices have changed everything [8]. The average amount of screen time has remained relatively constant since 2011, but mobile media use in children 0–8 years old has tripled since 2011 from 5 min to 15 min in 2013, and it has tripled again to 48 min per day in 2017 [9]. Currently, the average two-year-old uses a mobile device daily. Children of all ages are more likely to co-view with adults or anyone else when engaging with TV as compared to engaging with a tablet or smartphone [8]. For children 0–8 years old, 33% of all screen time is on a mobile device.

**Figure 1:**
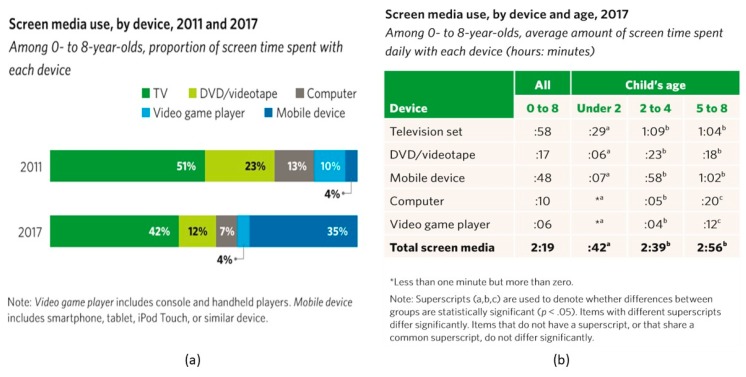
Screen Media use by device: (**a**) portion of screen time spent with each device in 2011 and 2017; (**b**) average amount of screen time spent daily with each device (hours:minutes).

Over 50% of parents whose children use screen media believe that it helps their child’s learning (Figure 1) [9]. There are a few studies that have illustrated the benefit of high-quality educational programs with the specific goal to teach academic skills, but only for children older than 2 years [10].

## 2. Review of the Literature

### 2.1. Potential Biological Effects of Early Screen Exposure

Exposing children to screens at an early age has the potential to become an addictive behavior. Growing up in a media-driven world will affect every generation differently. Nearly 50% of American teenagers who own a smartphone feel that they are addicted to their cell phones [11]. Today’s children are being inundated with unnatural levels of sensory stimulation at younger and younger ages. The fast-paced world that children’s programming portrays cannot compare to the real world, which is putting their fragile rewards system at an increased risk of corruption [12]. The main culprit is the neurotransmitter dopamine which floods the brain when we engage in screen time. In natural amounts dopamine is associated with learning, memory, motivation and pleasure. It rewards us for engaging in novel and stimulating activity, but as the amount of screen time consumption increases the more the brain becomes desensitized to dopamine. This results in needing an increasing amount of screen time to experience the same amount of pleasure [13].

Early screen exposure has several implications on the developing child. The brain develops most significantly in the first three years of life, which means that during those years the brain is at its most vulnerable stage of development [14,15]. Both longitudinal and cross-sectional studies have found that toddlers who engage in more than the recommended two hours of daily screen exposure had increased odds of low communication scores [16]. When children age 8–16 months view baby DVDs/videos there is a large negative association with vocabulary acquisition [17]. The daily amount of time a child spends engaging with various forms of media is not the only concern. The types of programming are equally as important. Six-month-old infants who were exposed to older child/adult-oriented content had lower levels of language and cognitive development at 14 months old [18]. Background television also has negative consequences, it has also been linked to reduced performance on cognitive tasks [19].

The link between traditional television and language development has been well established, but with 42% of children 0–8 years old having their own tablets and 95% of families having a smartphone, the impact of these new technologies needs to be considered [9]. Ma has recently showed that infants with more handheld screen time have an increased risk of an expressive speech delay [20]. An earlier age of media use onset and greater cumulative hours of media use are both significant independent predictors of poor executive functioning in preschoolers [21]. The effects of early screen exposure extend well into elementary school. For every additional hour of television exposure at 29 months there is a 7% and 6% unit decrease in classroom engagement and math achievement in the fourth grade [22].

### 2.2. Early Screen Exposure and Sleep

Early screen exposure impacts every aspect of a child’s life, their waking hours as well as the hours when they should be asleep growing and developing. Sleep deprivation manifests itself differently in children than in adults; children may face difficulty interacting with their peers, or may feel impulsive, sad, depressed, angry, or have mood swings [23]. Poor sleep habits negatively impact a child’s ability to perform well in the classroom [24]. Sleep is important for everyone, but especially for a developing child. Somatotropin, also known as human growth hormone, stimulates cell reproduction and growth. Most of this hormone is released during sleep, particularly in slow wave N3 stage [25]. Infants who slept longer/had better-quality nighttime sleep were longer/taller at their six-month check-up compared to infants with poorer sleep patterns [26]. Touchet et al. has shown that children who had short sleep durations in their first 2.5 years of life had increased cognitive defects and hyperactivity, even though they began and maintained a more normative sleep pattern from age 3–6 years old [27].

The increase of electronic media use has paralleled the decline in sleep time for children [28]. For every additional hour of television viewing, on average children slept seven less minutes per night and for every additional hour of tablet usage they slept sixteen minutes less. Children are sleeping less, and they are having increased difficulty falling asleep [29,30]. Excessive exposure to media throughout the day impacts sleep habits, but infants exposed to screen media in the evening hours show significantly shorter nighttime sleep duration than those with no evening screen exposure [31].

Excessive screen exposure contributes to decreased sleep time and increased daytime sleepiness through an array of mechanisms. One hypothesis is that the use of electronic media directly displaces sleep [32]. The type of programming that is being consumed can also impact sleep patterns. Garrison et al. has shown that there is an association between watching violent daytime media and shorter sleep duration [33]. Other mechanisms include the suppression of melatonin by short-wavelength (blue) light from the electronic devices, resulting in disrupted circadian rhythms and delayed sleep onset [32,34]. Another possibility is that excessive emotional and mental stimulation from electronic media use trigger a state of psychological and physiological hyper-arousal at bedtime in adolescents [28,32,35].

### 2.3. Early Screen Exposure and Obesity

Energy imbalance or consuming more calories from food and beverages than is used by the body for physical activity and growth results in weight gain [36]. Unfortunately, America is facing an obesity epidemic; approximately 20% of children and adolescents 2–19 are obese [37]. The percentage of obese children has more than tripled since 1970 [38]. Childhood obesity has far-reaching physical and emotional health implications. Some immediate concerns for obese children include an increased risk for other chronic conditions such as sleep apnea, type II diabetes, asthma, bone and joint problems, and risk factors for heart disease [36,39,40]. Obese children have a 54% higher rate of absenteeism from school than their normal weight counterparts and have poorer levels of academic achievement [41,42]. Furthermore, individuals who suffered from obesity as children are more likely to be obese adults [43]. Obesity in adulthood is associated with sleep apnea, heart disease, and an array of cancers [44,45,46].

The link between television viewing and obesity has been extensively researched for over 30 years [47]. The more TV children consume, the more likely they are to be obese. Adolescents who consume more than five hours of daily media are five times more likely to be overweight than their peers who consume 0–2 h [48]. Researchers have hypothesized that three different mechanisms play a role: increased caloric intake while engaging with TV or caused by advertising, reduced resting metabolism, and displacement of physical activity [49]. Consuming excessive amounts of media may displace sleep. Individuals with diminished sleep duration also experienced reduced levels of leptin and increased levels of ghrelin. This hormonal misbalance is likely to result in an increased appetite and may explain the elevated BMI (body mass index) that is observed with short sleep duration [50]. Watching TV for even more than 1.5 h daily is a risk factor for obesity [51]. For every hour of TV consumed by 2-year-olds, their BMI increases [52]. Pagani et al. found that for every additional hour of TV exposure at 29 months there was a 13% decrease in physical activity and a 5% increase in BMI [22]. Consuming any amount of TV appears to have potentially negative consequences. Kindergarteners who watched more than an hour of TV were 52% more likely to be overweight compared to their peers [53].

## 3. Discussion

### 3.1. Current Recommendations

Throughout the years, the American Academy of Pediatrics has changed their recommendations on how much quality programming children should engage in. Thanks to studies such as Peck et al., they currently recommend that children 2–4 engage in less than 1 h of quality programming daily (Table 1) [7,53].

### 3.2. Effects of Early Screen Exposure

**Figure 2:**
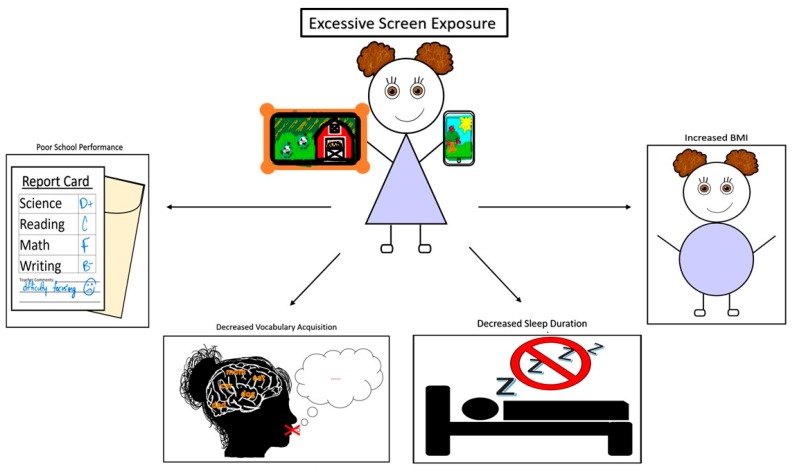
Effects of excessive screen exposure.

Children are being exposed to screens at earlier and earlier ages and we are just now elucidating the far-reaching effects of such technology. There is no benefit for children younger than 18 months to engage in screen time. They lack the ability to learn from and apply the 2-dimensional information to their 3-dimensional world, but they can become stimulated and potentially desensitized, leading to them requiring more screen time, for the same amount of pleasure, the older they get. The first three years of life are crucial for brain development. Several studies have enumerated the detrimental effects that early screen exposure can have on a child’s early life. Developing addictive behaviors and poor coping mechanisms at an early age can follow a child into adulthood and impact their health, earning potential, and social interactions (Figure 2).

### 3.3. Future Direction

Indulging in more than the recommended amount of screen time leads to poor sleep hygiene which can affect BMI, cognitive development, and academic performance. Screen time no longer means plopping down in front of the television in the family room. Children now have access to more screens and media than ever before, in their room as a TV, in their backpacks as tablets, in their hands as their caregiver’s or their own smartphones. Research on the impact of handheld devices compared to traditional television viewing still needs to catch up with the ever-changing technology-centric world that predominates today. It is possible for the American pediatric population to avoid a digital epidemic. Schools could get involved by tasking students to put down their technology one day a week. Parents need to monitor and regulate their child’s access to screens of all types and physicians need to inquire, educate, and help families develop media use plans. We are facing an environmental threat that has grown exponentially in past years and is projected to expand further in the future parallel to technology development. Despite solid evidence of the deleterious effects of this exposure in children, we are lacking epidemiological studies that inform about this environmental threat, which has hindered efforts in regulation and primary prevention. Further research is needed to develop a diagnostic that will reliably evaluate a child’s susceptibility to screen exposure and its far-reaching consequences.

## Figures and Tables

**Table 1:** Current daily screen time recommendations 0–5 years [7].

Age	American Academy of Pediatrics Recommendation
0–18 months	No screen time except video chatting
18–24 months	High quality programming/apps with active adult interaction
2–5 years old	One hour of quality programming/apps co-viewed with an adult
No screens during meals	
No screens at least 1 h before bedtime	
Turn off television and other devices when not in use	
